# An economic incentive package to support the wellbeing of caregivers of adolescents living with HIV during the COVID-19 pandemic in South Africa: a feasibility study protocol for a pilot randomised trial

**DOI:** 10.1186/s40814-023-01237-x

**Published:** 2023-01-09

**Authors:** Stanley Carries, Zibuyisile Mkhwanazi, Lovemore Sigwadhi, Mosa Moshabela, Makandwe Nyirenda, Jane Goudge, Darshini Govindasamy

**Affiliations:** 1 Health Systems Research Unit, South African Medical Research Council, Cape Town, South Africa; 2 Biostatistics Unit, Stellenbosch University, Stellenbosch, South Africa; 3 School of Nursing and Public Health, University of KwaZulu-Natal, Durban, South Africa; 4 Burden of Disease Unit, South African Medical Research Council, Cape Town, South Africa; 5 Centre for Health Policy, University of the Witwatersrand, Johannesburg, South Africa

**Keywords:** Caregivers, Wellbeing, HIV/AIDS, Economic incentives, Mental health, COVID-19, Sub-Saharan Africa

## Abstract

**Background:**

The mental and financial strain linked to unpaid caregiving has been amplified during the COVID-19 pandemic. In sub-Saharan Africa, carers of adolescents living with HIV (ALHIV) are critical for maintenance of optimum HIV treatment outcomes. However, the ability of caregivers to provide quality care to ALHIV is undermined by their ability to maintain their own wellbeing due to multiple factors (viz. poverty, stigma, lack of access to social support services) which have been exacerbated by the COVID-19 pandemic. Economic incentives, such as cash incentives combined with SMS reminders, have been shown to improve wellbeing. However, there is a lack of preliminary evidence on the potential of economic incentives to promote caregiver wellbeing in this setting, particularly in the context of a pandemic. This protocol outlines the design of a parallel-group pilot randomised trial comparing the feasibility and preliminary effectiveness of an economic incentive package versus a control for improving caregiver wellbeing.

**Methods:**

Caregivers of ALHIV will be recruited from public-sector HIV clinics in the south of the eThekwini municipality, KwaZulu-Natal, South Africa. Participants will be randomly assigned to one of the following groups: (i) the intervention group (*n* = 50) will receive three cash payments (of ZAR 350, approximately 23 USD), coupled with a positive wellbeing message over a 3-month period; (ii) the control group (*n* = 50) will receive a standard message encouraging linkage to health services. Participants will be interviewed at baseline and at endline (12 weeks) to collect socio-demographic, food insecurity, health status, mental health (stigma, depressive symptoms) and wellbeing data. The primary outcome measure, caregiver wellbeing, will be measured using the CarerQoL instrument. A qualitative study will be conducted alongside the main trial to understand participant views on participation in the trial and their feedback on study activities.

**Discussion:**

This study will provide scientific direction for the design of a larger randomised controlled trial exploring the effects of an economic incentive for improving caregiver wellbeing. The feasibility of conducting study activities and delivering the intervention remotely in the context of a pandemic will also be provided.

**Trial registration:**

PACTR202203585402090. Registry name: Pan African Clinical Trials Registry (PACTR); URL: https://pactr.samrc.ac.za/; Registration. date: 24 March 2022 (retrospectively registered); Date first participant enrolled: 03 November 2021

**Supplementary Information:**

The online version contains supplementary material available at 10.1186/s40814-023-01237-x.

## Background

Improving caregiver wellbeing in the post-COVID-19 context has become a public health priority. The pandemic has interrupted progress towards a key policy goal for carers and especially older persons (SDG 3: improving health and wellbeing for all ages) [1]. The mental and financial strain linked to this unpaid role is linked to negative health outcomes [2, 3] and economic consequences such as potential reduction in human capital accumulation and productivity losses [4]. Sub-Saharan Africa (SSA), with an estimated 1.54 million adolescents living with HIV (ALHIV), constitutes 88% of the global population of the ALHIV [5]. Despite remarkable progress in the scale-up of antiretroviral therapy (ART) in SSA, several studies report poor ART adherence, virological non-suppression [6] and high levels of depressive symptoms among this population [7]. A major predictor of favourable HIV and mental health outcomes among ALHIV is quality caregiving, which entails ensuring that the basic needs of adolescents are met (e.g. shelter, adequate nutrition, education) and providing emotional support through key milestones as they transition from childhood to adolescence (i.e. disclosure, self-acceptance and belonging, pubertal development, coping, relationship building) [8, 9]. However, caregivers are hampered in their ability to provide adequate support to ALHIV because of the barriers they encounter in maintaining wellbeing which are influenced by determinants that extend beyond the individual [10] such as gender inequalities and norms, stigma, lack of family or social support [11], household, socio-economic status [12, 13], geographic constraints in accessing health and social services [14]. The COVID-19 pandemic has further amplified these social determinants of wellbeing [15, 16]. Understanding which interventions are effective and feasible for improving caregiver wellbeing is an important first step for assessing how best to improve the quality of life among carers and ALHIV.

Most caregivers in SSA are older persons or females who are outside of the labour market and residing in skipped generation households that are at high risk of becoming impoverished [17, 18]. The gendered caregiving role intersects with poverty and other demographic, cultural and socio-political factors, (old age, diet, roles in in the household, education, access to basic health and social services, stigma) [19, 20]. Older carers from lower socio-economic status, living in rural areas with poor access to health and social services, and those that are HIV affected are likely to experience more stigma, severe mental health issues and hence, lower levels of wellbeing [21]. Evidence from SSA shows that caregivers’ fears around the consequences of inadvertent disclosure of adolescents’ HIV-positive status as well as their own, financial hardships, multiple caregiving responsibilities; and the lack of social support services are associated with pronounced negative effects on their mental health status (e.g. anxiety levels and depressive symptoms) and overall wellbeing (social isolation, purpose in life) [22–24]. The challenges with caregiving were further exacerbated by the COVID-19 pandemic, with caregivers experiencing adverse health, psychosocial and financial outcomes due to increased duties and caregiver burden [25] arising from disruptions of normal external support services [26]. It has also been reported that family caregivers demonstrated poorer mental health and physical health during the pandemic compared to non-caregivers [27].

In addition to tending to the special needs of ALHIV (e.g. adherence, clinic visits, transport costs, nutrition) [28, 29], primary caregivers are also challenged with looking after the basic needs of entire households [29–31]. Elderly caregivers, for instance, often stretch state pension grants to meet these needs [31]. Oftentimes caregivers have to resort to borrowing or selling essential resources to cope with their economic burden [29]. These households are thus economically vulnerable and the children living in them have the heightened risk of being malnourished [30].

Key barriers to caregiver wellbeing include lack of financial resources to support and care for ALHIV, attending to their personal care needs and household responsibilities, as well as fear of stigma [31]. Previous studies have shown that caregiver wellbeing in this setting is grounded in one’s ability to fulfil role-relationships [32, 33]. Several trials have shown that financial incentives are effective in improving HIV testing, including adherence to treatment and clinic appointments [34, 35]. Furthermore, financial incentives have been linked to improvements in quality of life [36]. Individuals are generally present-biased, loss-averse and ascribe high weights to low probability events [37]. Financial incentives may overcome these biases by offsetting the immediate opportunity costs linked to health-behaviour change along with uncertain and delayed gains [38]. Cash incentives for caregivers could potentially improve their quality of life by providing the resources to meet their personal care needs and the fiscal resources needed for them to plan and meet their caregiver and household responsibilities [31, 39]. More importantly, they could serve as recognition for the indispensable care they provide, thereby intensifying their sense of responsibility and quality of care provided to ALHIV.

Nudges are a key behavioural economics strategy (“nudge”) for health-related behaviour change [40, 41]. Nudges seek to influence decision-making by specifically targeting behavioural barriers. Nudges have been successfully used to develop low-cost solutions targeting key behavioural barriers and social norms impacting health outcomes [42]. Whilst there are mixed results on the effectiveness of nudges in certain settings, nudges have shown positive results in areas such as mental health [43, 44], HIV [45–47] and dietary choices [48]. In SSA, short-text messaging service (SMS) technology has been used in nudge approaches to deliver health information, motivate individuals to access care and adhere to treatment [47]. These studies also suggest that nudges can be readily brought to scale in these settings. Evidence from SSA indicates that SMS reminders sent to caregivers have been effective in promoting appointment attendance and medical adherence [47]. Key debates regarding the nudge approach revolve around its inability to solve complex health problems given its focus on targeting specific behavioural barriers when it comes to uptake, adherence and access to health interventions [49], the lack of causal evidence and lack of understanding of patterns [50, 51]. Ethical concerns noted about nudging are that it diminishes one’s autonomy and agency [51]. To our knowledge, no study has re-designed and evaluated these economic incentives, cash and SMS reminders, for the promotion of quality of life among caregivers of ALHIV.

Pilot and feasibility studies are an important first step in adapting and testing complex interventions in public health [52]. Pilot studies facilitate in-depth understanding of the conduct and applicability of an intervention which can enhance the design and conduct when evaluated at scale [53]. The overall aim of this pilot randomised control trial (RCT) is to evaluate the feasibility of a future large scale randomised controlled trial in examining the effectiveness of an economic incentive package (cash + SMS) in improving wellbeing among caregivers of ALHIV.

### Primary objectives

- To estimate the difference in wellbeing scores at baseline versus the endline among caregivers in the intervention versus control arm
- To ascertain the percentage of potential participants that both meet the eligibility criteria and enrol in the study
- To determine the participant recruitment and retention rate
- To establish compliance with the intervention process
- Piloting the methodological procedures, including randomisation, telephonic interviews, electronic data collection and electronic intervention delivery
- To reflect on the intervention protocol and amend as needed
- To describe participants’ views on acceptability by exploring their experiences of participating in the trial, assessing trial processes (content, delivery, utilisation, safety), and outcome measures

### Secondary objectives

- To examine the relationship between stigma, food insecurity and wellbeing
- To understand experiences of caregiver wellbeing and whether or not economic incentives shaped these experiences
- To estimate the total cost of delivery of the economic incentive and the average cost per person with an increase in caregiver wellbeing score in the intervention versus control arm
- Conduct a power calculation to determine the numbers needed for a large-scale RCT

## Methods

**Fig. 1:**
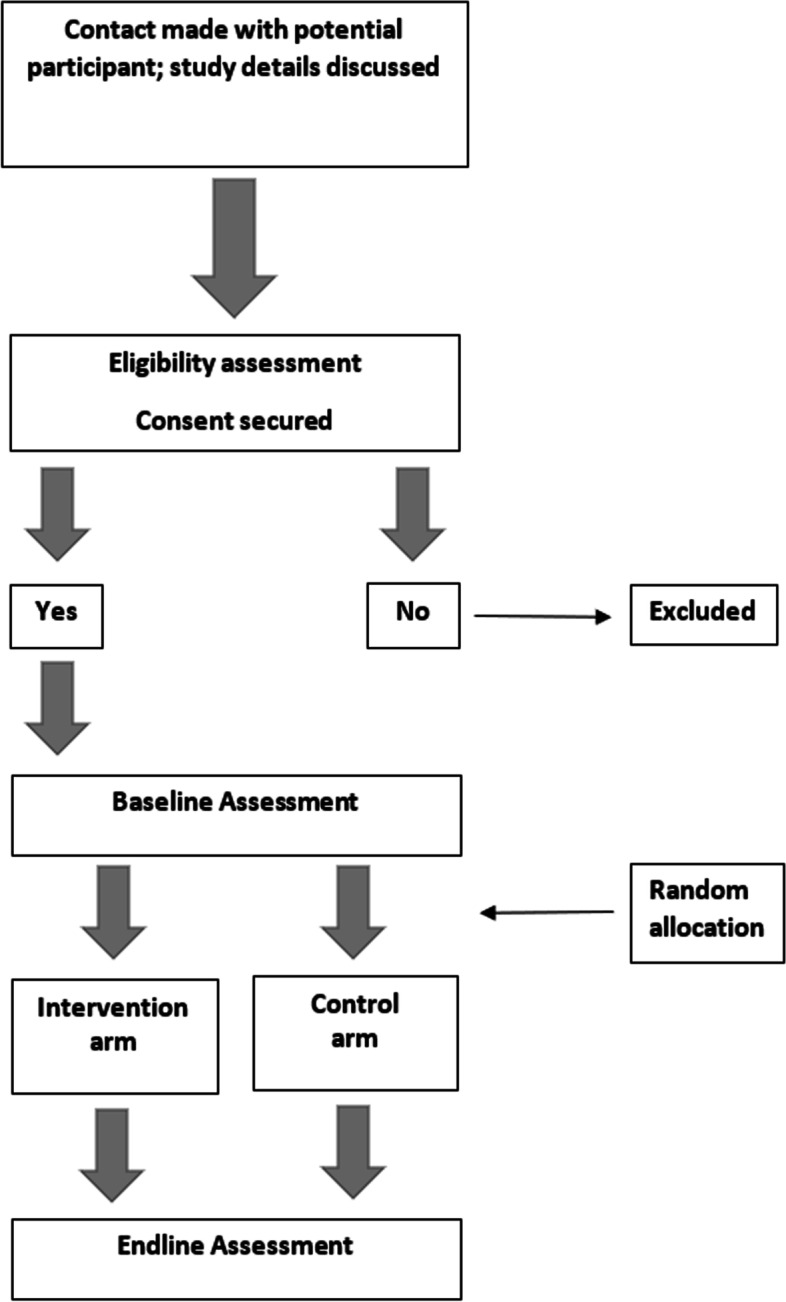
Standard protocol items: recommendation for intervention trials (SPIRIT) flow diagram

Reporting and methodology for the proposed study follow the Standard Protocol Items Recommendations for Interventional Trials (SPIRIT) [54] (see Fig. 1), as well as Consolidated Standards of Reporting Trials (CONSORT)—extension to randomised pilot and feasibility trials [55] (Additional file 4: Appendix 4). The intervention has been reported using the Template for Intervention Description and Replication (TIDieR) [56].

### Design

This will be a two-group parallel, open label pilot RCT. The study will have two arms, an intervention group (*n* = 50) and a control group (*n* = 50).

### Setting

This study will be conducted in a peri-urban community within the south of the eThekwini municipality, KwaZulu-Natal, South Africa. Recruitment, baseline quantitative assessments, and qualitative interviews will be conducted face-to-face. Whereas intervention delivery and end of trial assessments will be conducted telephonically.

### Participant recruitment

Participants will be recruited via:

1. An existing study database of caregivers of ALHIV who were enrolled in an adolescent study
2. Information sessions within local HIV clinics

### Participant eligibility criteria

Participants will be eligible to participate in the trial if they meet the following criteria:

- Caregivers (≥ 18 years of age) of ALHIV aged 10–19 years
- Caregivers who have their own personal mobile phone to ensure that there are no unauthorised cash withdrawals as the pin for retrieving the cash will be delivered to their mobile phone.

Participants will be excluded from the study should they have any of the following:

- Caregiver who is unable to comprehend the nature of the study during the information session in either English or isiZulu
- A caregiver who is experiencing distress, suicidal ideation, or requires urgent medical attention
- A caregiver who *does not* have their own personal mobile phone

### Screening

Initial contact will be face-to-face at the study clinic. Participant eligibility will be assessed, followed by explanation of the study, assessment of study understanding, and completion of consent procedures (Additional file 1: Appendix 1).

### Baseline and endline assessment

Post-screening, a baseline assessment will be conducted (Additional file 2: Appendix 2). After the intervention period, the endline assessment will be conducted. For the assessments, the following data will be collected on an electronic interviewer-administered REDCAP questionnaire:

1. *Socio-economic status* (e.g. demographics, dwelling type, household occupants, household dwellers’ ages), labour and income (e.g. employment status, income range), and household and social outcomes (e.g. number of children on support grants, source(s) of income).
2. *Food security* will be assessed using the Food Insecurity Experience Scale (FIES). The FIES has demonstrated good internal reliability in this setting (Rasch reliability infit statistic > 0.7) [57].
3. *Depressive symptoms* will be measured using the 10-item Centre for Epidemiological Studies Depression Scale (CES-D-10). The CES-D-10 has been psychometrically evaluated in a South African sample and showed acceptable internal consistency across different language groups (Cronbach’s α = 0.69–0.89), and concurrent validity when compared to other depressive symptom measures commonly used (e.g. Patient Health Questionnaire, WHO Disability Assessment Schedule) [58]. The scale has also shown good convergent validity (regression co-efficients between known psychosocial measures and EDS ranged from 0.17 to 0.19, *p* < 0.001) [59] and internal reliability (Cronbach’s α = 0.86) [60]. A cut-off point of 12 is deemed optimal to correctly classify individuals with a diagnosis of depression for a South African sample [61].
4. *Stigma:* the Everyday Discrimination Scale is a recommended scale for measuring intersectional stigma, particularly among people living with HIV [62], and has demonstrated good psychometric properties in samples from the United States of America [63].

#### Primary clinical outcome measures informing our sample size

1. Percentage change in wellbeing scores pre-vs. post-intervention:
  *Wellbeing* will be measured using the Care-related Quality of Life (CarerQol) instrument. The CarerQol instrument is a preference-based measure that was developed for use in economic evaluations [64], and has exhibited moderate construct validity (correlation co-efficient with CarerQol and self-rated burden dimensions ranging from 0.2 to 0.4) among older carers in the Netherlands [65]. Clinical and convergent validity have also been supported [66].
2. Successful consent rate: a minimum of ≥ 80% of eligible participants enrolled in the study
3. Successful retention rate: a retention rate of ≥ 80% of recruited participants

### Randomisation and allocation concealment

A statistician on the research team who is not involved in the intervention or outcome measure assessments will randomly allocate participants to the intervention or control group. The randomisation list will be generated in STATA (Stata Corp, College Station, TX, V17) using block randomisation techniques. This method is used to ensure a sample size balance across groups over time. Blocks will be small and balanced with predetermined group assignments, which keeps the numbers of subjects in each group similar. A seed will be used for the reproducibility of the same randomisation output obtained. Block randomisation with randomly selected block sizes of 2, 4, 8, and 12 will be generated. Four blocks of equal size (*n* = 25) will be generated in STATA, with two blocks assigned to each study arm (i.e. intervention: n=2 × 25 = 50; control: *n* = 25 × 2 = 50) and the list exported to Excel. This list will be emailed to a second researcher who will assign the intervention or control programme to each participant.

### Blinding

This is an open label trial as field staff will be involved in the intervention delivery and study assessments. The statistician involved in the randomisation will be blinded to study arm. Participants will be encouraged not to disclose details regarding their wellbeing programme to other participants during the trial. We will at multiple points (consent, baseline interview, cash send, follow-up) encourage participants not to share their study details, including randomisation, to anyone else.

### Intervention

Participants in the intervention group will receive an economic incentive package comprising a monthly motivational SMS and cash to the value of ZAR 350 (23 USD) for three months. The SMS will promote key behavioural economic principles (aspiration framing, loss aversion, altruism), aligned to social wellbeing dimensions that matter to people in this context (Table 1) [67]. The R350 incentive amount was chosen because it is within the margin of the COVID-19 Social Relief of Distress (SRD) grants in South Africa [68]. Participants will have 30 days to claim the receipt of their cash. It will be delivered via electronic local banking services. In line with the human centred design (HCD) approach, themed nudge messages will be co-developed with a caregiver advisory board (CAB), revised and used by the research team to design SMS messages to be sent to participant’s mobile phone via SMS by designated research team members using a standardised message template. The CAB will comprise 16 caregivers of ALHIV, who will not be part of the trial but from study community. The role of the CAB will include to inform study design, advising on intervention implementation, participant retention strategies, reviewing key findings, assisting with interpretation and dissemination of findings.

**Table 1:** Intervention messages and underpinning behavioural economic principle

Message	Behavioural economic principle	Message content
1	Aspiration framing	“Taking care of your own needs is important so you can be there to watch your child grow”
2	Loss aversion	“If you need to speak to someone you can call the South African Depression and Anxiety Group at no cost to you (Number:)”
3	Altruism	“Taking care of your child and family’s needs are important”

### Control

The control group will receive a once-off SMS encouraging access to public health clinic services as per the standard message sent by the Department Health in South Africa to patients accessing clinic services. Participants in this group will not receive cash or a motivational SMS.

### Qualitative study

A subset of participants in the intervention (*n* = 8) and control (*n* = 8) arm will be invited to participate in in-depth interviews after their baseline and endline quantitative assessments. They will be purposively sampled to ensure the sample is reflective of age, socio-economic status and HIV status. The aim of these IDIs is to provide an authentic account of wellbeing experiences among caregivers in this setting [69], to understand how experiences differ by arm and what could explain these differences. Topic guides (Additional file 3: Appendix 3) will be mapped on dimensions from our wellbeing scales (e.g. financial problems, relational problems, household and caregiving chores, mental and physical health, belonging) and preliminary findings from the trial. For those in the intervention arm, specific elements regarding the intervention will be probed as part of endline in-depth interview (i.e. timing, cash incentive amount, delivery method, safety concerns, SMS content, utilisation of the cash incentive, access of mental health support, recommendations to improve the intervention).

### Sample size

Our primary outcome is pre-post between difference in caregiver wellbeing scores. Assuming a mean caregiver wellbeing VAS score of 6.5 in M1 versus 8.5 in M5 (SD1.9) in the intervention arm, we would need a minimum sample size of *n* = 15 per arm to have 80% power to detect a treatment effect of between δ = 0.6–0.5 [66, 70]. Thus a sample size of *N* = 100 will satisfy this primary outcome and give us enough sample to look at feasibility measures (consent and retention rate ≥ 80%), assuming *n* = 20 refuse to participate or are lost to follow-up.

### Statistical analysis

Descriptive statistics will be used to present feasibility indicators and baseline sample characteristics at M1, including means (standard deviations) for normal data and, median (interquartile range) for non-normal data Differences in baseline characteristics between the intervention versus control arm, stratified by age-range of ALHIV (10–14 years. vs. 15–19 years) will be assessed using a chi-square test for categorical variables and Kruskal-Wallis *H* test for continuous variables. Baseline variables that differ between groups will be included as covariates in the regression analysis. To compare primary outcomes, we will analyse wellbeing score change at M1 and M5 between arms using linear regression. Furthermore, we will explore changes in scores between M2 and M5. To include all participants in the primary analysis (intention to treat analysis), we will perform multiple imputation methods if the loss of those missing from follow-up is significant, and assumptions are met. Participants who complete the questionnaire at baseline (M1) and endline (M5) will be included as per protocol. All statistical analyses will be performed using Stata (Stata Corp, College Station, TX, V17) (see Table 2 for participant timeline).

**Table 2:**
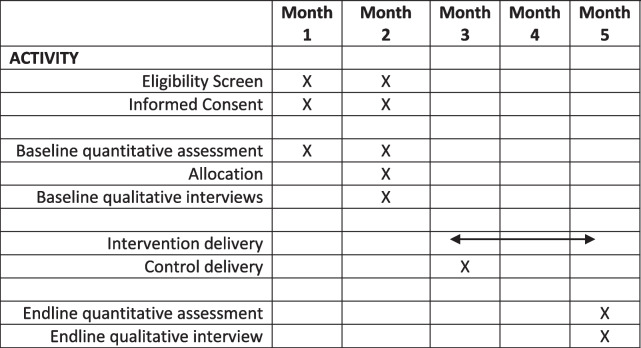
Participant timeline

### Progression criteria

The following criteria must be met in order to consider progression to a main RCT:

- A retention rate of ≥ 80% of recruited participants
- A minimum of ≥ 80% of eligible participants enrolled in the study

## Discussion

The aim of this pilot randomised trial is to compare the feasibility and preliminary effectiveness of an economic incentive package (cash + motivational SMS) versus a control for improving caregiver wellbeing. package. The results from this pilot trial will provide the critical data to determine whether a larger-scale RCT is warranted and feasible. This intervention represents an integrated approach, specifically designed to target aspects that matter to caregiver’s wellbeing in this setting (such as financial problems, mental health, relational problems, support, physical health) [14]. It is designed to be delivered remotely by lay counsellors and thus allows access to care for a vulnerable group who otherwise may not be able to receive such care due to time, transport limitations, lack of state psycho-social support services, along with the national social limitations implemented due to the COVID-19 pandemic.

Through this intervention we will investigate whether a cash incentive of three cash payments (of ZAR 350, approximately 23 USD) over a 3-month period, coupled with positive or affirmatory short messages, will have a positive influence on caregiver wellbeing. Several trials have shown that financial incentives are effective in improving HIV testing, including adherence to treatment and clinic appointments [34, 35], and have been linked to improvements in quality of life [36]. Offering a financial incentive may also help in overcoming caregiver present- and loss-averse biases by offsetting immediate opportunity costs linked to health-behaviour change, and uncertain and delayed gains [37, 38]. Additionally, the cash incentives could potentially improve their quality of life by providing them with resources to meet their personal care needs and the monetary resources needed to plan and meet their caregiver and household responsibilities [31, 39]. More importantly, it could serve as recognition for the indispensable care they provide, thereby intensifying their sense of responsibility and quality of care provided to ALHIV. The SMSs that will accompany each cash payment will serve as a nudge to deliver health information and to motivate caregivers to access care and adhere to treatment [45, 46]. Text messages have been shown to be effective in reducing depressive symptoms [43] and promoting uptake of mental healthcare [44]. Furthermore, studies in SSA have shown that SMS reminders sent to caregivers are effective in promoting appointment attendance and medical adherence [47].

In the context of high unemployment and lack of access to psycho-social support services, which have heightened during COVID-19 [25, 26, 71], our intervention could potentially show substantial improvement in carers wellbeing compared to the control arm. Alternatively, our results could show no effect as the COVID-19 pandemic is likely to have had substantial impact on carers mental health and wellbeing [25, 26, 71], requiring a more intensive intervention, targeting multiple levels (individual, household, community). Key ethical concerns of this trial include the need for carers to cover transport costs to commute to a cash withdrawal point to obtain the incentive, safety and security risks on the carer given the dangers of withdrawing cash in high crime informal areas and storing cash within households exposing them to potential GBV and substance abuse. Other ethical considerations include that the motivational SMSs could potentially induce emotional distress and depression as they could make carers more aware of their living conditions and poor mental health status. The loaning or sharing of the cash incentive between carers and delivery of the intervention to carers admitted to hospital or who have passed on could be a key challenge the trial could face.

The RCT will utilise a pre-post study design with a control group, with qualitative and cost data collected alongside the main trial data, which will be used to assess the end user acceptability and the affordability of the intervention. Data from this pilot trial will be used to inform the adaptation of the intervention and evaluation for a larger trial, which will provide robust evidence on the effectiveness and cost-effectiveness of this intervention [72]. We will thus be able to draw on the longitudinal qualitative data to help explain changes in our quantitative measures over time and compare changes to the control group. The intervention draws on the nudge approach and targets three behavioural barriers often targeted by nudges used in health programmes (i.e. aspiration framing, loss aversion and altruism). Furthermore, our evaluation of the intervention addresses some of the current shortcomings of the nudge approach in that it is using a causal design and drawing on qualitative research methods to further explain the patterns observed. Moreover, the cash component of the nudge seeks to address the food and economic insecurity of the household, a key social determinant of wellbeing. This intervention, if further assessed in a larger trial, could provide critical evidence for informing COVID-19 social relief policies which in many parts of SSA have centred around provision of households with cash and very little attention on mental health support. It could provide the data needed to advocate for cash + care programmes for carers as part of future pandemic preparedness.

### Limitations

The trial will be subject to the following limitations: (1) the sample will comprise a select group of caregivers consecutively drawn from an existing study database and approached during clinic appointments, limiting the generalisability of our findings; (2) whilst the wellbeing and other key measures have exhibited good psychometric properties, the isiZulu versions of these scales are yet to be robustly evaluated for this setting; (3) it is highly probable that the cash incentive value may be insufficient to impact wellbeing. However, the value of the cash incentive is higher than that used in previous studies which have shown promising effects on HIV care outcomes; (4) the duration of the intervention may be too short for individuals to change behaviours and make major shifts in their wellbeing; (5) a two-armed trial (cash + SMS versus control), does not allow us to specifically examine the effects of individual intervention components on wellbeing. However, the qualitative study will probe specific elements of the intervention, and this will provide some idea on how each component facilitated wellbeing; (6) participants will not be blinded to trial arm. However, participants will be encouraged not to disclose their programme randomisation status to other participants at each stage of contact (i.e. consent, baseline interview, cash send delivery, follow-up interview); (7) although study sample size was sufficient to generate 80% power to detect treatment effects, this sample size might not be sufficient to extrapolate statistical analysis results that are generalisable over the entire ALHIV caregiver population; and (8) the lack of blinding could add bias to findings. It is, therefore, recommended that future full-scale trials use a cluster RCT approach to minimise bias.

### Trial status

Enrolment of participants and intervention and control arm delivery complete. Baseline and follow-up interviews complete. Analysis currently underway.

## Supplementary Information

**Additional file 1: Appendix 1.** Informed Voluntary Consent Form.

**Additional file 2: Appendix 2.** Baseline and exit questionnaire-pilot RCT.

**Additional file 3: Appendix 3.** Topic guide-qualitative interviews (IDIs).

**Additional file 4.** CONSORT 2010 checklist of information to include when reporting a randomised trial*.

## Abbreviations

ALHIV: Adolescents living with HIV
CAB: Caregiver advisory board
HCD: Human centred design
HSRU: Health systems research unit
RCT: Randomised control trial
SAMRC: South African Medical Research Council
SDG: Sustainable Development Goals
SMS: Short message service
SSA: Sub-Saharan Africa
