# Supplementary material for: An economic incentive package to support the wellbeing of caregivers of adolescents living with HIV during the COVID-19 pandemic in South Africa: a feasibility study protocol for a pilot randomised trial
Source: Pilot Feasibility Stud. 2023 Jan 9;9:3. doi: 10.1186/s40814-023-01237-x (PMC9827020; doi:10.1186/s40814-023-01237-x)
Supplement: Supplementary file 1 — Additional file 1: Appendix 1. Informed Voluntary Consent Form. [file 40814_2023_1237_MOESM1_ESM.docx]

## **Appendix 1**: Informed Voluntary Consent Form

Please indicate whether you agree to taking part in this study or not by marking the boxes provided with an **X** in the table below. If you agree to taking part in the study, please kindly add your name, signature and date in the spaces provided below the table.

| **Please read and mark each statement below** | **Please mark with an X** | |
| --- | --- | --- |
| - I confirm that I have read and understood the information sheet for the above study and have had the opportunity to ask questions. | - Yes | - No |
| - I have received a copy of the information sheet in my preferred language. | - Yes | - No |
| - I understand that my consent is voluntary and that I am free to withdraw this consent at any time, without giving any reason and without my legal rights being affected. | - Yes | - No |
| **Overall, I agree to taking part in this study** |  |  |
| - I agree to participate in this study no matter which study arm that I am enrolled into. | - Yes | - No |
| - I agree to quotes or other results arising from what I say in this study being included in any reports, as long as I cannot be identified. | - Yes | - No |
| - I agree to share my contact details with the study staff should they need to contact me to discuss any study matters. | - Yes | - No |
| **As per the Protection of Personal Information Act 4 of 2013 (as amended), I agree:** | | |
| - To personal information (data) from me being collected, processed, shared and stored as per the research protocol approved by South African Medical Research Council's Human Research Ethics Committee | - Yes | - No |
| - To data that does not contain my name(s) being shared, processed and transferred to other researchers in or outside South Africa | - Yes | - No |
| - To results arising from what I say in this study being included in any reports, as long as I cannot be identified | - Yes | - No |

| **For participant to complete:** |  |  | |
| --- | --- | --- | --- |
| **______________________________**  Name and surname of participant | **___________________________**  Signature or initials | **____________________________**  Today’s date (dd/mm/yyyy) | |
|  |  |  | |
|  |  |  | |
|  |  |  | |
| **For witness to complete if participant is unable to write:**  *As a witness I confirm that all information about this study was given and the participant verbally consented to taking part* | | |  |
| **______________________________**  Name and surname of witness | **___________________________**  Signature | **___________________________**  Today’s date (dd/mm/yyyy) |  |

| **For study staff to complete:** |  |  |
| --- | --- | --- |
| *I declare that I have explained the information given in this document to the participant and the participant was given ample time to ask questions* | | |
| **______________________________**  Name and surname of staff taking consent | **___________________________**  Signature | **___________________________**  Today’s date (dd/mm/yyyy) |
