# Supplementary material for: An economic incentive package to support the wellbeing of caregivers of adolescents living with HIV during the COVID-19 pandemic in South Africa: a feasibility study protocol for a pilot randomised trial
Source: Pilot Feasibility Stud. 2023 Jan 9;9:3. doi: 10.1186/s40814-023-01237-x (PMC9827020; doi:10.1186/s40814-023-01237-x)
Supplement: Supplementary file 2 — Additional file 2: Appendix 2. Baseline and exit questionnaire-pilot RCT. [file 40814_2023_1237_MOESM2_ESM.docx]

## Appendix 2- Baseline and exit questionnaire- pilot RCT

**Socio-economic Status**

1. **Demographic Information**

| **Question** | **Response** | |
| --- | --- | --- |
| 1. How old are you? (baseline only) | Age: _______________ | |
|  | Don’t know | |
|  | Refused | |
| 1. What is your date of birth? (dd MMM yyyy) (baseline only) | __________________  dd / mm/ yyyy | |
| 1. What is your gender? (baseline only) | Male | |
|  | Female | |
|  | Other *(Specify below)*  ________________ | |
|  | Refused | |
| 1. What is your race? (baseline only) | Black/African | |
|  | Indian/Asian | |
|  | Coloured | |
|  | White | |
|  | Other *(Specify below)*  ________________ | |
|  | Refused | |
| 1. What is the highest grade that you have completed or passed? | _________________ | |
| 1. Have you successfully completed any tertiary qualifications like diplomas, certificates or degrees? | Yes | |
|  | No | |
| 1. Do you have a partner or are you married (including traditional marriage) | Yes | |
|  | No | |
| 1. If you have a partner or are married, do you currently live with them? | Yes | |
|  | No | |
| 1. What type of dwelling or house do you live in? | House/flat | |
|  | Traditional house (e.g. mud house) | |
|  | Informal house (e.g. shack) | |
|  | Don’t know | |
| 1. Do you have piped or tap water inside your dwelling or house or in your yard? | Yes | |
|  | No | |
| 1. Does your dwelling or house have access to electricity? | Yes | |
|  | No | |
| 1. Do you currently have any of the following conditions? | - TB - Asthma - Diabetes - Hypertension - HIV - Cancer - COVID-19 - Arthritis | - Kidney disease - Heart condition - Other:____ |
| 1. How many people (including yourself) live in the household? ***(Don’t forget to include babies).*** | _________________ | |
| 1. How many are younger than 5 years old? | _________________ | |
| 1. How many are between 5 and 18 years old? | _________________ | |
| 1. How many are between 18 and 60 years old? | _________________ | |
| 1. How many are 60 years or older? | _________________ | |

1. **Labour and Household Economic Status**

| **Question** | **Response** |
| --- | --- |
| 1. What is your employment status? | Employed |
|  | Self-employed |
|  | Temp/casual worker |
|  | Do odd jobs |
|  | Unemployed ***(skip to 3)*** |
| 1. If you get some sort of income, can you please indicate approximately how much you earn in a month? |  |
|  |  |
| ***(skip to 5 after answering)*** | _________________ |
|  |  |
|  | Refused |
|  |  |
|  |  |
| 1. If you are not working, what is the main reason for this? | COVID-19 Lockdown related |
|  | Poor health |
|  | Retrenched |
|  | Retired/too old/pensioner |
|  | Pregnant/maternity |
|  | Disability |
|  | Have never worked |
|  | Other *(Specify below)*  ________________ |
|  | Refused |
| 1. If you worked before, but are now no longer working, when was the last time that you worked? | Less than 3 months ago |
|  | 3 to 6 months ago |
|  | 6 to 9 months ago |
|  | 9 to 12 months ago |
|  | more than a year ago |
| 1. How much money is spent every month by your household on: |  |
| 1. Food | **R**_________________ |
| 1. Transport   *(e.g. to work, school, to look for jobs, doing to hospital/clinic GP, etc.)* | **R**_________________ |
| 1. Healthcare-related products or services   *(e.g. visits to the doctor, hospital / clinic visits, medication, etc.)* | **R**_________________ |
| 1. Water | **R**_________________ |
| 1. Electricity | **R**_________________ |
| 1. Other important household things | **R**_________________ |
| 1. Can you please tell me what some of those other things are? |  |
|  |  |
|  |  |
|  |  |
|  |  |

1. **Household and Social Outcomes**

| **We would like to get a better understand of your household situation (e.g. the number of people living in your household, support from family, friends, government, etc.). Can you please answer the following questions about your household?** | |
| --- | --- |
| **Question** | **Response** |
| 1. How many child support grants does your household receive? | Number:___________ |
|  | Refused |
|  | Don’t know |
| 1. How many people in your household receive an old age pension grant? | Number:___________ |
|  | Refused |
|  | Don’t know |
| 1. Do you personally receive any kind of government grant? | Yes |
|  | No |
| 1. Which government grant (or grants) do you receive? | Child Support Grant |
|  | Old Age Pension Grant |
|  | Disability Grant |
|  | Foster Child Grant |
|  | Care Dependency Grant |
|  | Other *(Specify below)*  ________________ |
|  | Refused |
| 1. Did you receive any government grant on behalf of someone else? | Yes |
|  | No |
| 1. Do you or your household get any other form of income? | Yes |
|  | No |
| 1. Please indicate all the sources of income that your household receives. *(Please tick (√) all that apply)* | Income from employment |
|  | Income from business |
|  | Government grants |
|  | Money from friends and family |
|  | Pension |
|  | Other *(Specify below)*  ________________ |
|  | Refused |
| 1. In the past month, did you receive food or shelter from any of these sources? | Government |
|  | NGO’s, churches, and other associations |
|  | Neighbours or community |
|  | Other *(Please specify)*  *___________________* |
| 1. Do you frequently take any member of your family to a healthcare facility or collect medication for them? | Yes |
|  | No |
| 1. If yes, how much of your time was spent doing this in the past month? | less than 4 hours |
|  | 4 to 8 hours |
|  | 8 to 12 hours |
|  | More than 12 hours |

1. **Questions relating specifically to costs associated with taking care of ALHIV**

| **We would now like to get a better understanding of the costs that you have when looking after the health of your child over the past month.** | |
| --- | --- |
| 1. **Direct Costs:** | |
| **Question** | **Response** |
| **In the past month, how much money have you spent on…** |  |
| 1. Transport taking your child to and from health facilities (e.g. clinic, hospital, general practitioner, etc.)? | **R**: __________________ |
| 1. Admission and consultation fees? | **R**: __________________ |
| 1. Medication? | **R**: __________________ |
| 1. Food while at the health facility? | **R**: __________________ |
| 1. Any fees paid for diagnostic procedures? | **R**: __________________ |
| 1. Cost for somebody else accompanying your child to healthcare facility other than yourself? | **R**: __________________ |
| 1. Cost for somebody else taking care of your child when you are not around (e.g. at work)? | **R**: __________________ |

| 1. **Indirect Costs:** | |
| --- | --- |
| **Question** | **Response** |
| **In the past month…** |  |
| 1. How often were you unable to carry out your normal daily activities at all due to your child being ill (e.g. (in)formal work, doing chores, etc.)? | **Days:** __________________ |
| 1. How often were you unable to carry out your normal daily activities for a few hours due to your child being ill? | **Days:** __________________ |
| 1. **Coping Strategies:** | |
| **Question** | **Response** |
| **In the past month…** |  |
| 1. Can you please indicate how you have been able to cope with looking after the healthcare of your child | Borrowing interest free money |
|  | Borrowing interest baring money |
|  | Help from neighbours or community |
|  | Family labour substitution |
|  | Selling assets |
|  | Children stop going to school |
|  | Family contributions |
|  | Social grants |
|  | Other *(Please specify)*  *___________________* |

1. **Food Insecurity Experience Scale (FIES): Household Referenced**

| **Now I would like to ask you some questions about food. During the last 12 MONTHS, was there a time when: *(Please tick (√) your response to each statement in the spaces provided)*** | | | | | |
| --- | --- | --- | --- | --- | --- |
| **Statement** | **No** | **Yes** | | **Don’t know** | **Refused to answer** |
| 1. You or others in your household worried about not having enough food to eat because of a lack of money or other resources? |  |  | |  |  |
| 1. Thinking about the last 12 MONTHS, was there a time when you or others in your household were unable to eat healthy and nutritious food because of a lack of money or other resources? |  |  | |  |  |
| 1. Was there a time when you or others in your household ate only a few kinds of foods because of a lack of money or other resources? |  |  | |  |  |
| 1. Was there a time when you or others in your household had to skip a meal because there was not enough money or other resources to get food? |  |  | |  |  |
| 1. Still thinking about the last 12 MONTHS, was there a time when you or others in your household ate less than you thought you should because of a lack of money or other resources? |  |  | |  |  |
| 1. Was there a time when your household ran out of food because of a lack of money or other resources? |  |  | |  |  |
| 1. Was there a time when you or others in your household were hungry but did not eat because there was not enough money or other resources for food? |  |  | |  |  |
| 1. Was there a time when you or others in your household went without eating for a whole day because of a lack of money or other resources? |  |  | |  |  |
| **Please indicate what your feelings or thoughts are about the following:** | | | | | |
| **Questions** | | | **Responses** | | |
| 1. If you lost a wallet with R200 in it, how likely do you think that it will be returned by a stranger? | | | Very/fairly likely | | |
|  |  |  | Unlikely | | |
| 1. If you lost a wallet with R200 in it, how likely do you think that it will be returned by a neighbour? | | | Very/fairly likely | | |
|  |  |  | Unlikely | | |
| 1. How important is religion to you? | | | Very important /important | | |
|  |  |  | Unimportant / not important at all | | |

**Mental Health**

1. **Centre for Epidemiologic Studies Depression Scale (CES-D-10)**

| Below is a list of the ways you might have felt or behaved. Please tell me how often you have felt this way during the past week by ticking the appropriate box to each statement. | | | | | |
| --- | --- | --- | --- | --- | --- |
|  | **During the Past Week** | | | |  |
|  | Rarely or none of the time (less than 1 day) | Some or a little of the time (1-2  days) | Occasionally or a moderate amount of time (3-4 days) | Most or all of the time (5-7 days) |  |
| 1. I was bothered by things that usually don’t bother me. | 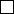 | 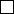 | 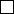 | 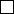 |  |
| 1. I had trouble keeping my mind on what I was doing. | 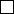 | 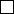 | 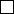 | 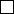 |  |
| 1. I felt depressed. | 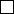 | 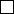 | 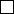 | 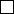 |  |
| 1. I felt that everything I did was an effort. | 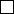 | 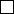 | 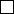 | 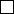 |  |
| 1. I felt hopeful about the future. | 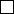 | 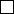 | 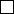 | 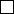 |  |
| 1. I felt fearful. | 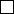 | 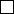 | 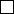 | 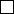 |  |
| 1. My sleep was restless. | 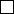 | 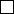 | 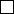 | 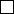 |  |
| 1. I was happy. | 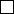 | 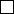 | 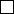 | 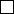 |  |
| 1. I felt lonely. | 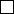 | 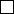 | 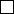 | 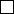 |  |
| 1. I could not “get going.” | 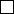 | 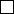 | 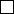 | 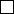 |  |

1. **Everyday Discrimination Scale (Expanded)**

| **In your day-to-day life, how often do any of the following things happen to you?**  ***(Please tick (√) next to each statement the one response that best describes how often you experiences)*** | | | | | | |
| --- | --- | --- | --- | --- | --- | --- |
| **Statement** | **Almost everyday** | **At least once a week** | **A few times a month** | **A few times a year** | **Less than once a year** | **Never** |
| 1. You are treated with less courtesy than other people are. |  |  |  |  |  |  |
| 1. You are treated with less respect than other people are. |  |  |  |  |  |  |
| 1. You receive poorer service than other people at restaurants or stores. |  |  |  |  |  |  |
| 1. People act as if they think you are not smart. |  |  |  |  |  |  |
| 1. People act as if they are afraid of you. |  |  |  |  |  |  |
| 1. People act as if they think you are dishonest. |  |  |  |  |  |  |
| 1. People act as if they’re better than you are. |  |  |  |  |  |  |
| 1. You are called names or insulted. |  |  |  |  |  |  |
| 1. You are threatened or harassed. |  |  |  |  |  |  |
| 1. You are followed around in stores. |  |  |  |  |  |  |

**Wellbeing**

**H: CareQol-7-D Scale**

1. **Mental Health Continuum - Short Form**

| **Please answer the following questions are about how you have been feeling during the past month, past. Place a check mark in the box that best represents how often you have experienced or felt the following:** | | | | | | |
| --- | --- | --- | --- | --- | --- | --- |
| **During the past month how often did you feel …** | **Never** | **Once or twice** | **About once a week** | **About 2 to 3 times a week** | **Almost every day** | **Every day** |
| 1. happy |  |  |  |  |  |  |
| 2. interested in life |  |  |  |  |  |  |
| 3. satisfied |  |  |  |  |  |  |
| 4. that you had something important to contribute to society |  |  |  |  |  |  |
| 5. that you belonged to a community (like a social group, or your neighborhood) |  |  |  |  |  |  |
| 6. that our society is becoming a better place for people like you |  |  |  |  |  |  |
| 7. that people are basically good |  |  |  |  |  |  |
| 8. that the way our society works makes sense to you |  |  |  |  |  |  |
| 9. that you liked most parts of your personality |  |  |  |  |  |  |
| 10. good at managing the responsibilities of your daily life |  |  |  |  |  |  |
| 11. that you had warm and trusting relationships with others |  |  |  |  |  |  |
| 12. that you had experiences that challenged you to grow and become a better person |  |  |  |  |  |  |
| 13. confident to think or express your own ideas and opinions |  |  |  |  |  |  |
| 14. that your life has a sense of direction or meaning to it |  |  |  |  |  |  |
