# Supplementary material for: An economic incentive package to support the wellbeing of caregivers of adolescents living with HIV during the COVID-19 pandemic in South Africa: a feasibility study protocol for a pilot randomised trial
Source: Pilot Feasibility Stud. 2023 Jan 9;9:3. doi: 10.1186/s40814-023-01237-x (PMC9827020; doi:10.1186/s40814-023-01237-x)
Supplement: Supplementary file 3 — Additional file 3: Appendix 3. Topic guide-qualitative interviews (IDIs). [file 40814_2023_1237_MOESM3_ESM.docx]

## Appendix 3- Topic guide- qualitative interviews (IDIs)

**Interview questions**

*At the beginning of the interview, usher the participant into the conversation by first talking about themselves. Ask them about their background etc. Try to get the participant to talk about their life in such a way that will easily flow with the interview topic areas*

**USHERING PARTICIPANT INTO THE CONVERSATION:**

- Tell me more about yourself.
- Where are you originally from?

1. **CAREGIVER WELLBEING QUESTIONS:**
2. **Environment**

We would like to form an impression of your caregiving situation. These questions will help us understand what your caregiving situation is like right now.

1. Can you describe your current caregiving role?

***Probe the following with regarding to caregiving***

- 1. number of individuals caring for (age, conditions, sex, length of time, key responsibilities)
  2. financial costs
  3. key challenges
  4. support received
  5. emotional burden (stress, fatigue, sleeping patterns, depression, anxiety)
  6. physical health challenges

1. **Mental Health**

We are now going to talk about your emotions (feelings)

1. What makes you feel happy?
2. What makes you feel sad?

**Probe the following:** Caregiving, relationships, support, finances, loneliness, isolation, sleep patterns, fear, anxiety, internalised/experienced stigma coping strategies

1. **INTERVENTION-RELATED**

We would now like to know a little bit about the intervention that you received over the past 3 months. This intervention was made up of two parts. In the first part we sent you SMSs to help promote your wellbeing. In the second part we sent you money via cashsend.

1. **Promoting caregiver well-being SMS intervention**

*Let’s begin talking about the SMSs that were sent to you to promote your wellbeing:*

1. What were your thoughts on the messages in the SMSs that we sent to you?
   1. ***Probe:*** likes/dislikes, frequency, preferred methods of communication
2. How could we improve getting messages across to you and other caregivers in the future?
3. **Cash incentive intervention**

*Now, can you please tell me about the cashsend intervention:*

1. Did you find the money part of the intervention helpful?
2. Please tell me about the timing of the intervention. Was the cash sent at a time when you really needed it?
3. Do you think that the incentive amount was a reasonable amount?
   1. Can you please explain why you feel this way?
4. Did you have any problems with the money being sent to you via cashsend?
   1. Can you please explain?
5. Did you have any problems with getting to an ATM to withdraw the money?
   1. What were some of the problems that you had?
6. Did you have any safety concerns about going to withdraw the money from an ATM?
7. What is your overall impression about how the money was sent to you?
8. Can you please tell us how you spent your money?
9. Can you suggest ways that we could improve getting the money to you or other caregivers in the future?
